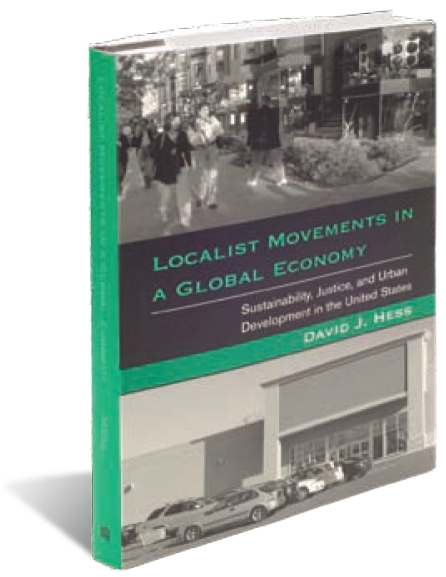# Localist Movements in a Global Economy: Sustainability, Justice, and Urban Development in the United States

**Published:** 2009-12

**Authors:** Anthony J McMichael

**Affiliations:** Anthony J McMichael holds an Australia Fellowship at the National Centre for Epidemiology and Population Health, Australian National University. His research interests are in environmental health, including climate change, urbanism, food, and sustainability

The title of this book clearly echoes the well-known exhortation of microbiologist Rene Dubos: “Think global, act local.” We live today under the broad and darkening shadow of large-scale disruptions by human societies to the natural environment and its life-support systems. We also face an increasingly urban future as the still-expanding human population gravitates into cities in most countries around the world. This book examines the reawakened hopes being pinned on the rise of local stewardship of environmental resources, particularly within the urban context, and the associated localization of economic relations and activities.

Further impetus and credibility will presumably accrue to localist movements from the awarding of the 2009 Nobel Prize for Economics to Elinor Ostrom. Her work has shown that, when communities are given the opportunity to work together and share equitably in the management of local environmental resources, then Garrett Hardin’s notion of the “tragedy of the commons” is not inevitable. Indeed, free-riding “theft” of an extra personal share of environmental resources becomes unlikely. Ostrom has concluded from wide-ranging evidence that the productivity and sustainability of that collectivist approach work better than when government or the private sector controls the process (which, further, is usually done on a larger scale).

The recent growth of localist movements, in both high- and low-income countries, has occurred against the background of an increasingly globalized economy, an economy that is widely experienced as distant, “corporate,” depersonalized, inequitable, and beyond local influence. During the past decade or so in the United States, dozens of local business organizations with local, community-based roots have arisen. Typically, these initiatives have sought to build local ownership into key industries such as retail, food, energy, transportation, and media. The underlying stimulus and challenge is the emerging felt need to restore the architecture of economic and political ownership for rural, urban, and other subna-tional regions. This, therefore, is a welcome book that both describes and evaluates this localist phenomenon within the U.S. context—and pays particular attention to the overwhelming contemporary challenges of achieving both social justice and environmental sustainability.

David Hess is realistic about the globalized economy, which (notwithstanding its many adverse, often inequitable, outcomes for humans and environments) is with us for the foreseeable future. That global economy can, he argues, be leavened, supplemented, and humanized via the growth of localism. However, the benefits of localism are not assured. Via his analysis of various case studies, conducted within a broad interdisciplinary frame, Hess teases out the features of localism that best accord with the achievement of sustainability, social justice, and, thus, enhanced urban development. Chapter 3, “Can Localism be Just and Sustainable?” provides revealing examples of where the answer is sometimes yes, sometimes no. This includes an engaging discussion of the well-known formulations about “food-miles”: Importing seasonally grown vegetables may be less environmentally damaging than the unseasonal, energy-subsidized, local production of those same foods.

Other chapters explore the politics of local retailing, the challenges of urban agriculture, the relationship of local energy production to the public sector, and the infusion of localism into the media industry. Hess brings these analyses together in a final chapter, “Policies for an Alternative Economy,” wherein he echoes the first chapter by examining the possibilities of localist solutions to global problems.

The localist movement faces, of course, formidable inertia in and opposition from the established corporatist economy. This issue has been touched on elsewhere in David Owen’s related book *Green Metropolis: Why Living Smaller, Living Closer, and Driving Less are the Keys to Sustainability* (Riverhead, 2009). Owen focuses particularly on life in Manhattan, and his tract is strong in describing the manifestations of the general environmental crisis of today, and underlining the enormity and urgency of the resultant challenge to our ways of living in cities. Hess, though, provides a more scholarly analysis of the forms of that challenge, and whether and how we can find a better (more democratic, just, and sustainable) way of living in cities. His book provides insights into the workings and consequences of several particular domains of localism: “buy local” campaigns, urban agriculture, local ownership of electricity and transportation, and alternative and community media.

This is a thoughtful and well-researched book—as is evident from the end notes for each chapter and from the very extensive bibliography. The writing is clear, and the arguments are well presented. Hess is supportive of but not starry-eyed about the role and prospects for localism. It is not, he judges, *the* solution; rather, it is an important part of the solution. He concludes with these measured words: “As the collapse scenarios of the twenty-first century unfold, the world will need all the partial solutions and all the resilience it can muster.”

## Figures and Tables

**Figure:**